# Safety and tolerability of AZD5363 in Japanese patients with advanced solid tumors

**DOI:** 10.1007/s00280-016-2987-9

**Published:** 2016-03-01

**Authors:** Kenji Tamura, Jun Hashimoto, Yuko Tanabe, Makoto Kodaira, Kan Yonemori, Takashi Seto, Fumihiko Hirai, Shuji Arita, Gouji Toyokawa, Lan Chen, Hiroshi Yamamoto, Toshio Kawata, Justin Lindemann, Taito Esaki

**Affiliations:** Department of Breast and Medical Oncology, National Cancer Center Hospital, 5-1-1, Tsukiji, Chuo-ku, Tokyo, 104-0045 Japan; Department of Thoracic Oncology, National Hospital Organization Kyushu Cancer Center, Fukuoka, Japan; Department of Gastrointestinal and Medical Oncology, National Hospital Organization Kyushu Cancer Center, Fukuoka, Japan; Research and Development, AstraZeneca KK, Osaka, Japan; AstraZeneca Innovative Medicines Unit, Cambridge, UK

**Keywords:** AZD5363, Akt inhibitor, Safety, Solid tumor, Akt1 (E17K) mutation

## Abstract

**Purpose:**

Investigate the safety and tolerability of AZD5363 and define a recommended dose for evaluation in Japanese patients with advanced solid malignancies.

**Methods:**

AZD5363 was administered orally as a single dose, and then the dose was escalated to twice daily (bid) in separate continuous (every day) and intermittent (4 days on, 3 days off [4/3] or 2 days on, 5 days off [2/5]) dosing schedules to reach recommended doses defined by dose-limiting toxicity (DLT). Doses for continuous, 4/3, and 2/5 intermittent dosing schedules were 80–400, 360–480, and 640 mg, respectively, and were informed by results from an equivalent study in Caucasian patients.

**Results:**

Forty-one patients received AZD5363. DLTs were only experienced with continuous dosing. 97.6 % of patients reported at least one adverse event (AE); most common were diarrhea (78.0 %), hyperglycemia (68.3 %), nausea (56.1 %), and maculopapular rash (56.1 %). Grade ≥3 AEs were reported by 63.4 % of patients. Exposure of AZD5363 was generally dose proportional for both single and multiple doses. Single-dose pharmacokinetics of AZD5363 was generally predictive of multiple-dose pharmacokinetics. Confirmed partial responses were reported by two patients, both of whom were Akt1 (E17K) mutation positive. One patient in the 480 mg bid 4/3 dosing cohort maintained partial response for >2 years.

**Conclusions:**

Intermittent dosing of AZD5363 was more tolerable than continuous dosing. 480 mg bid intermittent 4/3 dosing for AZD5363 monotherapy was selected for further investigation. Preliminary evidence of antitumor activity was observed. Akt1 (E17K) is a potent driver mutation that may predict clinical response to AZD5363.

## Introduction

The PI3K/Akt/mTOR signaling pathway is an attractive target for anticancer therapies given its crucial role in tumor progression [1]. Akt is an important signaling node within this pathway, promoting tumorigenesis and metastasis and inhibiting apoptosis [2]. Aberrant Akt signaling is associated with anticancer drug resistance, and inhibition of Akt is known to confer susceptibility to various treatment modalities, such as chemotherapy and radiotherapy [3, 4]. To date, monotherapeutic interventions targeting the PI3 K/Akt/mTOR pathway have been generally unsuccessful [5], likely because of incomplete target inhibition or compensatory feedback loops that lead to drug resistance [5]. More effective inhibitors of the PI3K/Akt/mTOR pathway administered to appropriately selected patients or in more biologically relevant combinations are required.

AZD5363, a potent, selective inhibitor of Akt, has demonstrated antitumor activity by overcoming or delaying resistance to pharmacological therapies in several cancer xenograft models [6]. A first inpatient study has evaluated AZD5363 administered to Western patients with advanced solid malignancies (AstraZeneca study D3610C00001, NCT01226316) [7]. Patients received escalating doses of AZD5363 in separate dosing regimens, either continuous twice daily (bid) every day or intermittent (4 days on, 3 days off [4/3]), in order to establish the maximum tolerated dose (MTD). From these studies, a recommended dose of 480 mg bid was determined for AZD5363 monotherapy in a 4/3 dosing schedule.

Objectives of the current study were to investigate the safety and tolerability of AZD5363 and to define a recommended dose for further clinical evaluation when given either continuously (bid dosing every day) or intermittently (4/3 or 2 days on, 5 days off [2/5]) to Japanese patients with advanced solid malignancies. This study is registered with ClinicalTrials.gov, number NCT01353781 (AstraZeneca study D3610C00004).

## Methods

### Patients

Patients aged ≥20 years with histological or cytological confirmation of a solid malignant tumor refractory to standard therapies or with no suitable effective standard treatments, as well as a World Health Organization (WHO) performance status of 0–1, were eligible for study inclusion.

The study was approved by an independent ethics committee and complied with the International Conference of Harmonisation Harmonised Tripartite Guidelines for Good Clinical Practice, the Declaration of Helsinki, local laws, and the AstraZeneca policy on bioethics [8]. All patients provided written informed consent.

### Study design and treatment

This was a phase 1, open-label, multicenter, dose escalation study. For the first cohort, each patient received a single 80-mg dose of AZD5363 within 7 days of screening (cycle 0) followed by 3–7 days of washout. All doses were required to be taken in a fasted state (no caloric intake from 2 h before dosing to 1 h after dosing). A continuous dosing regimen (cycle 1) was then initiated, with patients receiving AZD5363 bid every day.

All evaluations during the bid dosing regimen were conducted in 21-day assessment cycles. Patients were enrolled to ensure a minimum of three and a maximum of six evaluable patients per cohort. Dose escalation and de-escalation were determined by the number of dose-limiting toxicities (DLTs; defined as any toxicity related to study drug as assessed by the investigator and not attributable to the disease under investigation) in a cohort. If no DLT was observed in a cohort of 3–6 evaluable patients, the dose could be escalated. If one patient in a group of >3 evaluable patients experienced a DLT, the cohort was expanded to include six evaluable patients. If only one DLT was observed in the complete cohort of six evaluable patients, the dose could then be escalated. If ≥2 patients in a group of up to six evaluable patients experienced a DLT, irrespective of the number of patients enrolled, the dose was considered not tolerated and recruitment to the cohort and dose escalation was ceased. De-escalation to a lower intermediary dose could be considered in order to better define the MTD.

Based on emerging clinical data from the continuous dosing regimen of this study and data from the Western Study 1 (D3610C00001, NCT01226316), which was running in parallel with the Japanese study, an intermittent dosing regimen was also evaluated in Japanese patients and with a starting dose of 480 mg bid and a 4/3 dosing schedule.

The primary objective of the study was to investigate the safety and tolerability of escalating doses of AZD5363 in order to define a recommended dose in Japanese patients with advanced solid tumors. Secondary objectives included characterizing the pharmacokinetics (PK) of AZD5363 and a preliminary assessment of the antitumor activity of AZD5363 monotherapy.

### Assessments

Safety and tolerability were assessed in terms of adverse events (AEs), serious adverse events (SAEs), deaths, laboratory data, vital signs, electrocardiogram (ECG), assessment of glucose metabolism, and physical examination. AEs were graded according to the Common Terminology Criteria for Adverse Events (CTCAE) version 4.0. Any AE occurring after the first dose of study treatment and within 30 days of the last dose of study treatment was included in the AE summaries.

For PK analyses, plasma concentrations of AZD5363 were assessed up to 48 h after a single dose. After multiple doses, plasma concentrations were assessed up to 12 h on days 2, 4, and 8 for the 2/5 and 4/3 intermittent dosing and continuous dosing schedules, respectively. Single-dose PK parameters assessed included area under the plasma concentration–time curve (AUC), maximum plasma (peak) drug concentration (*C*_max_), time to reach *C*_max_ (*t*_max_), apparent plasma clearance (CL/F), and oral volume of distribution at steady state (*V*_ss_/*F*). Steady-state PK parameters included AUC_ss_, *C*_ss,max_, *t*_ss,max_, and CL_ss_/*F*.

Objective tumor response assessments were based on Response Evaluation Criteria in Solid Tumors (RECIST) version 1.1.

Tumor samples were screened for mutations using a clinical sequencing platform consisting of an in-house cancer gene panel (National Cancer Center OncoPanel version 2) based on an Agilent Sure Select system and an Illumina MiSeq sequencer. The target regions to be captured were the exons of 90 targetable or actionable genes and the introns of 10 protein kinase genes. Samples with <10 % tumor cell content were excluded from the analysis.

### Statistical analyses

Data were summarized using descriptive statistics, and no formal statistical analyses of the primary or secondary endpoints were performed. Patients were grouped by schedule and dose. All calculations were performed with the SAS^®^ software, version 9.1.3 or higher.

The safety and PK analysis sets comprised all patients who received at least one dose of AZD5363 and all patients who provided concentration–time data for AZD5363, respectively.

## Results

### Patient characteristics

Fifty-two patients were enrolled in the study, of which 41, all of Japanese ethnicity, received AZD5363 and were evaluable for safety analyses. Of these patients, 39 discontinued study treatment (data cutoff July 7, 2014), 27 of whom did so as a result of worsening of the condition under investigation, with the remainder owing to patient decision to discontinue (*N* = 6), adverse events (*N* = 5), and protocol non-compliance (*N* = 1). There was one protocol deviation that was considered to be important (one patient did not fulfill eligibility criteria because of a familial history of long QT syndrome—this patient was withdrawn from the study on Day 9 of Cycle 0) in the 360 mg bid intermittent 4/3 dosing cohort.

The overall demographic and disease baseline characteristics of the study population were considered representative of the target population and are summarized in Table 1. There were 24 females (58.5 %) and 17 males (41.5 %), with a mean age of 52.0 years (range: 27–73 years). Breast cancer (19.5 %) was the most common primary tumor. With the exception of one patient, all had metastatic disease at baseline (97.6 %). Prior to study entry, all patients had received ≥1 chemotherapy regimen, while 78.1 % had received ≥3 chemotherapy regimens (median number of prior chemotherapy regimens was 4.0 [range: 1–8]).

**Table 1:** Patient demographics and baseline characteristics

	Continuous				Intermittent			
	80 mg bid	240 mg bid	320 mg bid	400 mg bid	360 mg bid 4/3	480 mg bid 4/3	640 mg bid 2/5	Total
*n*	3	7	6	5	8	6	6	41
Mean age (range) (years)	55.3 (37–65)	56.1 (43–72)	47.0 (33–63)	51.8 (36–67)	54.4 (38–73)	44.3 (27–64)	55.0 (41–62)	52.0 (27–73)
Males/females (*n*/*n*)	0/3	3/4	3/3	3/2	4/4	2/4	2/4	17/24
WHO performance status [*n* (%)]								
0	3 (100.0)	5 (71.4)	1 (16.7)	3 (60.0)	3 (37.5)	5 (83.3)	5 (83.3)	25 (61.0)
1	0 (0.0)	2 (28.6)	5 (83.3)	2 (40.0)	5 (62.5)	1 (16.7)	1 (16.7)	16 (39.0)
Primary tumor site [*n* (%)]								
Breast	1 (33.3)	1 (14.3)	2 (33.3)	0 (0.0)	2 (25.0)	0 (0.0)	2 (33.3)	8 (19.5)
Lung	0 (0.0)	0 (0.0)	1 (16.7)	1 (20.0)	1 (12.5)	1 (16.7)	1 (16.7)	5 (12.2)
Uterus	2 (66.7)	0 (0.0)	0 (0.0)	0 (0.0)	1 (12.5)	1 (16.7)	1 (16.7)	5 (12.2)
Pleura	0 (0.0)	0 (0.0)	1 (16.7)	0 (0.0)	2 (25.0)	0 (0.0)	0 (0.0)	3 (7.3)
Ovary	0 (0.0)	0 (0.0)	0 (0.0)	1 (20.0)	0 (0.0)	1 (16.7)	0 (0.0)	2 (4.9)
Liver	0 (0.0)	0 (0.0)	0 (0.0)	1 (20.0)	0 (0.0)	1 (16.7)	0 (0.0)	2 (4.9)
Colorectal^a^	0 (0.0)	2 (28.6)	0 (0.0)	0 (0.0)	1 (12.5)	1 (16.7)	0 (0.0)	4 (9.8)
Other^b^	0 (0.0)	4 (57.1)	2 (33.3)	2 (40.0)	1 (12.5)	1 (16.7)	2 (33.3)	12 (29.3)
Lines of previous chemotherapy [*n* (%)]								
1	0 (0.0)	1 (14.3)	0 (0.0)	0 (0.0)	0 (0.0)	2 (33.3)	0 (0.0)	1 (2.4)
2	1 (33.3)	0 (0.0)	0 (0.0)	1 (20.0)	3 (37.5)	18 (100)	1 (16.7)	8 (19.5)
≥3	2 (66.7)	6 (85.7)	6 (100.0)	4 (80.0)	5 (62.5)	4 (66.7)	5 (83.3)	32 (78.1)
Median	3.0	4.0	4.0	3.0	3.0	3.5	4.0	4.0

^a^Includes cecum and rectal

^b^Anterior mediastinum, duodenum, endometrium stomale, hypopharyngeal, leimyosarcoma, esophagus, pancreas, stomach, thymus, urinary bladder, unknown

### Safety and tolerability outcomes

In the safety analysis set, 97.6 % of patients experienced at least one AE, of which 95.1 % were judged by the local investigator to be related to AZD5363 (Table 2). Overall, the most frequently reported AEs were diarrhea (78.0 %), hyperglycemia (68.3 %), nausea (56.1 %), and maculopapular rash (56.1 %). A total of 63.4 % of patients experienced an AE of CTCAE grade ≥3, with hyperglycemia (39.0 %) and diarrhea (17.1 %) being the most common; 58.5 % of patients experienced a grade ≥3 AE considered to be treatment related. Discontinuations due to an AE occurred in 12.2 % of patients, while 39.0 % of patients had AEs leading to dose interruption. Three patients experienced an SAE, all judged by the local investigator to be unrelated to AZD5363. There were no deaths related to AEs.

**Table 2:** Summary of adverse events: number (%) of patients reporting at least one adverse event (frequency >35 %)

	Continuous				Intermittent			
	80 mg bid	240 mg bid	320 mg bid	400 mg bid	360 mg bid 4/3	480 mg bid 4/3	640 mg bid 2/5	Total
*n*	3	7	6	5	8	6	6	41
Adverse events (AEs)								
Any	3 (100.0)	6 (85.7)	6 (100.0)	5 (100.0)	8 (100.0)	6 (100.0)	6 (100.0)	40 (97.6)
Diarrhea	0 (0.0)	5 (71.4)	5 (83.3)	5 (100.0)	7 (87.5)	5 (83.3)	5 (83.3)	32 (78.0)
Hyperglycemia	0 (0.0)	3 (42.9)	4 (66.7)	5 (100.0)	5 (62.5)	5 (83.3)	6 (100.0)	28 (68.3)
Nausea	1 (33.3)	3 (42.9)	5 (83.3)	5 (100.0)	3 (37.5)	3 (50.0)	3 (50.0)	23 (56.1)
Maculopapular rash	0 (0.0)	2 (28.6)	4 (66.7)	5 (100.0)	3 (37.5)	6 (100.0)	3 (50.0)	23 (56.1)
Decreased appetite	0 (0.0)	2 (28.6)	4 (66.7)	4 (80.0)	5 (62.5)	2 (33.3)	3 (50.0)	20 (48.8)
Pyrexia	1 (33.3)	4 (57.1)	3 (50.0)	5 (100.0)	3 (37.5)	3 (50.0)	1 (16.7)	20 (48.8)
Stomatitis	0 (0.0)	3 (42.9)	3 (50.0)	4 (80.0)	3 (37.5)	3 (50.0)	1 (16.7)	17 (41.5)
Proteinuria	0 (0.0)	0 (0.0)	3 (50.0)	1 (20.0)	3 (37.5)	4 (66.7)	5 (83.3)	16 (39.0)
Blood creatinine increased	0 (0.0)	2 (28.6)	4 (66.7)	3 (60.0)	2 (25.0)	2 (33.3)	1 (16.7)	14 (34.1)
White blood cell count decreased	0 (0.0)	1 (14.3)	3 (50.0)	1 (20.0)	3 (37.5)	4 (66.7)	1 (16.7)	13 (31.7)
AEs causally related to AZD5363^a^								
Any	3 (100.0)	6 (85.7)	6 (100.0)	5 (100.0)	7 (87.5)	6 (100.0)	6 (100.0)	39 (95.1)
AEs of CTCAE grade ≥3								
Any	0 (0.0)	3 (42.9)	4 (66.7)	5 (100.0)	5 (62.5)	4 (66.7)	5 (83.3)	26 (63.4)
Hyperglycemia	0 (0.0)	2 (28.6)	2 (33.3)	3 (60.0)	1 (12.5)	3 (50.0)	5 (83.3)	16 (39.0)
Diarrhea	0 (0.0)	0 (0.0)	3 (50.0)	1 (20.0)	2 (25.0)	0 (0.0)	1 (16.7)	7 (17.1)
Neutrophil count decreased	0 (0.0)	0 (0.0)	0 (0.0)	0 (0.0)	3 (37.5)	0 (0.0)	1 (16.7)	4 (9.8)
Lymphocyte count decreased	0 (0.0)	1 (14.3)	1 (16.7)	1 (20.0)	0 (0.0)	0 (0.0)	0 (0.0)	3 (7.3)
AEs of CTCAE grade ≥3 causally related to AZD5363^a^								
Any	0 (0.0)	3 (42.9)	4 (66.7)	5 (100.0)	3 (37.5)	4 (66.7)	5 (83.3)	24 (58.5)
AE with outcome of death								
Any	0 (0.0)	0 (0.0)	0 (0.0)	0 (0.0)	0 (0.0)	0 (0.0)	0 (0.0)	0 (0.0)
SAE								
Any	0 (0.0)	0 (0.0)	0 (0.0)	0 (0.0)	0 (0.0)	1 (16.7)	2 (33.3)	3 (7.3)
SAE causally related to AZD5363^a^								
Any	0 (0.0)	0 (0.0)	0 (0.0)	0 (0.0)	0 (0.0)	0 (0.0)	0 (0.0)	0 (0.0)
AEs leading to discontinuation of AZD5363								
Any	0 (0.0)	1 (14.3)	1 (16.7)	1 (20.0)	0 (0.0)	1 (16.7)	1 (16.7)	5 (12.2)

^a^As assessed by the investigator

In the 480 mg bid intermittent 4/3 dosing cohort, the most common AEs were maculopapular rash (100.0 %), diarrhea (83.3 %), and hyperglycemia (83.3 %), with 66.7 % of patients experiencing at least one AE of CTCAE grade ≥3 [hyperglycemia was most frequent (50.0 % of patients across all the cohorts)]. One patient in this cohort discontinued treatment because of treatment-related AEs of rash and drug hypersensitivity, while another patient had an SAE of intra-abdominal hemorrhage, which was judged by the investigator to be unrelated to treatment.

Overall, there were no obvious trends for changes in laboratory parameters, vital signs, ECG parameters, or physical examinations compared with baseline during the study. One patient in the 360 mg bid 4/3 dosing cohort was observed to have elevated glycosylated hemoglobin [HbA_1c_; 5.9 % (baseline) to 8 % (cycle 4) and 7.5 % (cycle 10)], possibly as a result of mild anemia experienced by the patient throughout the study [9].

DLTs were experienced by five patients in the continuous dosing cohorts: one patient in the 240 mg bid cohort (grade 3 hypoxia—this event was considered by the investigator as possibly induced by treatment-related events of high fever and maculopapular rash; higher plasma concentrations were observed in this patient vs. other patients in this treatment cohort), two patients in the 320 mg bid treatment group (grade 3 diarrhea in both cases), and two patients receiving 400 mg bid (grade 3 diarrhea, and grade 3 oral mucositis and maculopapular rash). No DLTs were reported in any of the intermittent dosing cohorts.

### Pharmacokinetics

**Fig. 1:**
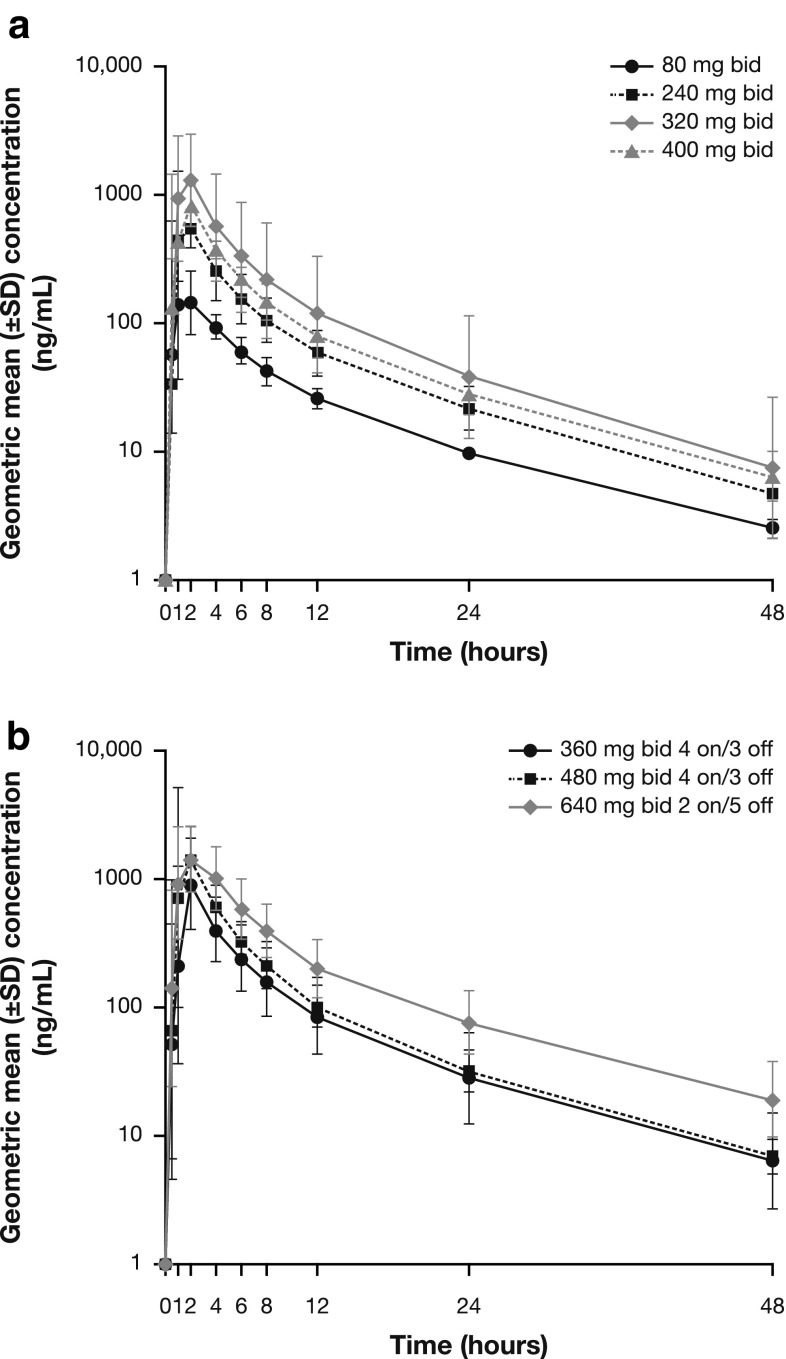
Plasma concentration–time profile of single-dose AZD5363 after **a** continuous dosing and **b** intermittent dosing

**Fig. 2:**
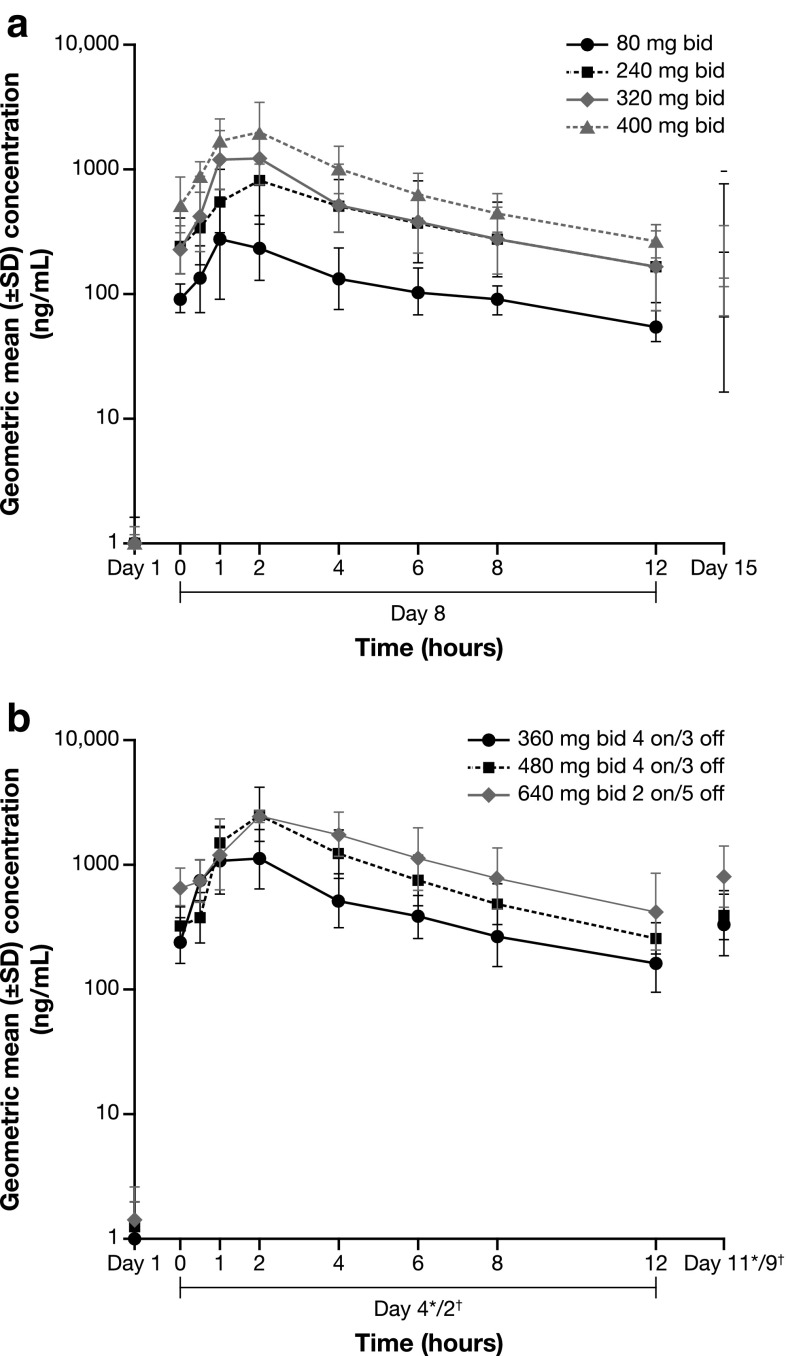
Plasma concentration–time profile after multiple doses (study day 8) of AZD5363 for **a** continuous dosing and **b** intermittent dosing. *360 mg bid 4 on/3 off and 480 mg bid 4 on/3off; ^†^640 mg bid 2 on/5 off

Following single oral doses, AZD5363 was quantifiable in all samples collected across the profile from each patient, with the exception of one patient (40 evaluable patients). Following *C*_max_, there was a noticeable distribution phase generally lasting up to 6–8 h, consistent with the half-life of AZD5363 of approximately 10 h, with an apparent terminal elimination phase established thereafter (Figs. 1, 2). Exposure data for both single and multiple doses of AZD5363 were generally dose proportional (Table 3). The rate of absorption following multiple dosing was considered to be moderate, with a median (range) *t*_ss,max_ of 1.98 (0.50–3.97) hours, which was consistent with single-dose *t*_max_ data of 1.98 (0.87–4.02) hours.

**Table 3:** Plasma PK parameters of AZD5363 after a single dose and multiple doses

	Continuous				Intermittent			Total
	80 mg bid	240 mg bid	320 mg bid	400 mg bid	360 mg bid 4/3	480 mg bid 4/3	640 mg bid 2/5	
*Single-dose AZD5363*								
*n*	3	7	6	5	8	6	6	41
AUC (ng h/mL)	1265 (26.3)	3293 (39.2)	7109 (115.5)	4666 (20.6)	4772 (69.2)	7208 (46.0)	11390 (41.5)	5184 (86.6)
*C*_max_ (ng/mL)	217 (101.2)	628 (40.8)	1582 (80.2)	1062 (27.9)	1008 (65.5)	1907 (84.7)	2099 (37.1)	1091 (96.3)
*t*_half_ (hours)	11.8 (8.5‒12.2)	10.2 (8.2‒10.9)	9.7 (7.3‒10.6)	9.6 (9.0‒13.0)	9.7 (5.3‒11.1)	9.3 (8.1‒11.6)	10.1 (9.4‒13.7)	9.9 (5.3‒13.7)
*t*_max_ (hours)	2 (1‒4)	1.9 (0.87‒2)	1.98 (0.98‒2.08)	1.97 (0.98‒2.17)	1.99 (0.98‒2.03)	1.025 (0.93‒1.98)	2.015 (1‒4.02)	1.98 (0.87‒4.02)
CL/F (L/h)	63.24 (26.3)	72.89 (39.2)	45.01 (115.5)	85.72 (20.6)	75.45 (69.2)	66.59 (46.0)	56.17 (41.5)	65.57 (57.7)
*V*_ss_/*F* (L)	715.1 (58.9)	682.4 (41.5)	370.2 (97.0)	777.0 (34.1)	680.7 (58.3)	502.2 (63.6)	587.1 (44.2)	594.7 (59.7)
*Multiple-dose AZD5363*								
*n*	3	7	4	4	7	6	6	37
AUC_ss_ (ng h/mL)	1518 (59.0)	4839 (77.2)	5772 (55.2)	9666 (43.7)	5866 (45.0)	10,930 (33.1)	14,210 (36.7)	6820 (86.0)
*C*_ss,max_ (ng/mL)	318.3 (128.4)	874.4 (89.7)	1222 (53.7)	2094 (56.6)	1366 (64.9)	2670 (36.7)	2618 (20.0)	1430 (96.6)
*t*_ss_,_max_ (hours)	1 (0.98‒1.97)	1.98 (0.97‒3.9)	1.465 (0.92‒2.12)	2 (0.93‒2)	0.98 (0.5‒2)	1.99 (1‒2.05)	2.01 (2‒3.97)	1.98 (0.5‒3.97)
CL_ss_/*F* (L/h)	52.72 (59.0)	49.6 (77.2)	55.44 (55.2)	41.38 (43.7)	61.37 (45.0)	43.9 (33.1)	45.04 (36.7)	49.71 (48.6)

Data are presented as geometric mean (CV%), apart from *t*_half_, *t*_max_ and *t*_ss,max_, which are presented as median (range)

### Antitumor activity

The tumor response analysis set included 37 patients with a baseline tumor assessment. The best objective response according to RECIST version 1.1 is summarized in Table 4. Confirmed partial responses were reported by two patients: one each in the 480 mg bid intermittent 4/3 and 640 mg bid intermittent 2/5 dosing cohorts. At data cutoff, the durations of partial response for these two patients were 675 and 127 days, respectively. Ten patients (three on continuous dosing and seven on intermittent dosing) had stable disease, with durations ranging from 46 to 360 days. Seven of these 10 patients had stable disease for 12 weeks or more. Disease progression was reported by 48.6 % of patients, and no patients had a complete response to AZD5363 treatment.

**Table 4:** Best objective response

	Continuous				Intermittent			Total
	80 mg bid	240 mg bid	320 mg bid	400 mg bid	360 mg bid 4/3	480 mg bid 4/3	640 mg bid 2/5	
*n*	3	7	6	5	8	6	6	41
Response								
Complete response^a^	0 (0.0)	0 (0.0)	0 (0.0)	0 (0.0)	0 (0.0)	0 (0.0)	0 (0.0)	0 (0.0)
Partial response^a^	0 (0.0)	0 (0.0)	0 (0.0)	0 (0.0)	0 (0.0)	1 (16.7)	1 (16.7)	2 (5.4)
Non-response								
Stable disease ≥6 weeks	2 (66.7)	1 (14.3)	0 (0.0)	0 (0.0)	3 (37.5)	4 (66.7)	0 (0.0)	10 (27.0)
RECIST progression	1 (33.3)	5 (71.4)	1 (33.3)	3 (75.0)	3 (37.5)	1 (16.7)	4 (66.7)	18 (48.6)
Not evaluable	0 (0.0)	1 (14.3)	2 (66.7)	1 (25.0)	2 (25.0)	0 (0.0)	1 (16.7)	7 (18.9)

^a^Response confirmed after 4 weeks

## Discussion

This phase 1 study was primarily designed to evaluate the safety and tolerability of AZD5363 when given at increasing doses and in continuous and intermittent dosing schedules to Japanese patients with advanced solid malignancies. Patients were assigned to receive AZD5363 either as continuous bid dosing (80–400 mg every day) or as intermittent bid dosing (360–480 mg for 4 days/week or 640 mg for 2 days/week) in 21-day cycles. Overall, five patients (all on continuous dosing) experienced DLTs. MTD was not reached in either of the intermittent dosing schedules in this study, but given the results from the equivalent Western study, no further dosing increase from the MTDs determined for intermittent doses from that study were conducted in this study. AEs of CTCAE grade ≥3 causally related to AZD5363 were more frequent in the 640 mg bid intermittent 2/5 dosing cohort than in the 480 mg bid intermittent 4/3 dosing cohort (any AE, 83.3 vs. 66.7 %; hyperglycemia, 83.3 vs. 50.0 %; diarrhea, 16.7 vs. 0.0 %; neutrophil count decreased, 16.7 vs. 0.0 %), indicating more favorable tolerability with 480 mg bid intermittent 4/3 dosing. Intermittent dosing has been advocated as an alternative to continuous dosing for oncology therapeutics to enable adequate pathway inhibition for efficacy with reduced toxicity or receptor reactivation[10].

The primary aim of the study was to determine the safety and tolerability of AZD5363 in the target patient population. Overall, 97.6 % of patients reported at least one AE, the majority (95.1 %) of which was considered treatment related. Increased rates of AEs were generally observed with increasing dose, irrespective of dosing regimen, while the frequency of AEs was generally similar across the dosing schedules. The most common AEs experienced by Japanese patients treated with AZD5363 were diarrhea (78.0 %), hyperglycemia (68.3 %), nausea (56.1 %), and maculopapular rash (56.1 %), which is consistent with data for AZD5363 in Western patients [7, 11]. Diarrhea resolved during and off AZD5363 treatment in 91 % of patients who experienced this AE (88 % received antidiarrheal treatment), while hyperglycemia appeared to be manageable through the use of oral metformin. Rash resolved in 58.5 % of patients during and off AZD5363 treatment. Hyperglycemia is an expected side effect of PI3K/AKT/mTOR pathway inhibition given the role of the pathway in regulating insulin signaling and glucose homeostasis [12, 13]. Maculopapular rash has been observed in clinical studies of other PI3 K/AKT/mTOR pathway inhibitors [14]. Although the pathophysiological mechanism of drug-induced maculopapular rash is unknown, pathway inhibition may induce local cytokine and chemokine deregulation, leading to cutaneous toxicity [5].

A secondary objective of the study was to characterize the PK of AZD5363. Analysis revealed that the rate of absorption following single or multiple bid doses of AZD5363 was moderate and appeared to be independent of dose. Plasma concentration–time profiles were similar across the different dosing schedules. Taking into account the small cohort sizes and data variability, the geometric mean for *C*_max_ and AUC data was generally dose proportional for the 80- to 640-mg doses. The PK half-life of AZD5363 was appropriate for administration by the intermittent dosing regimen. These findings are consistent with those of another phase 1 study evaluating AZD5363 in Western patients [7].

A further secondary objective was to assess the antitumor activity of AZD5363 by evaluation of tumor response using RECIST criteria. Confirmed partial responses were experienced by two patients receiving AZD5363. The first achieved a partial response 79 days after starting 480 mg bid intermittent 4/3 dosing of AZD5363. This 38-year-old female patient had primary ovarian cancer (well differentiated, T1N0M0 at diagnosis) with metastatic disease in the lung and had received eight prior treatment regimens. The patient has achieved continued maintenance of partial response (duration of response was 1177 days as of September 2015, according to the investigator), with a best response of a 55.8 % decrease in the sum of the longest diameter (SLD) of target lesions.

The second patient with long-duration disease control had primary estrogen-receptor-positive, HER2-negative papillary carcinoma breast cancer (non-assessable grade, T4N1MX at diagnosis, based on the patient report form) with metastatic disease in a mediastinal lymph node and the liver on study entry, and had received three chemotherapeutic regimens and six hormonal therapies prior to study entry. The patient, who was assigned to the 640 mg bid intermittent 2/5 dosing cohort, achieved a partial response at 44 days after starting AZD5363 therapy. However, progression was confirmed at Day 378, with a best response of a 60.8 % decrease in the SLD of target lesions.

The mutation status of both patients who achieved partial responses was analyzed retrospectively (response was prospectively investigated as a secondary objective) and reported by the investigator. For the first patient, prestudy tumor samples from lung metastases were revealed to be Akt1 (E17K) and CTNNB1 (G34V) mutation positive. Archival primary tumor tissue from the second patient harbored an Akt1 (E17K) and TP53 mutation, as well as amplification of the CCND1 gene. Analysis of archival primary tumor samples from the 37 patients in the responder analysis set showed that, of the non-responders, two additional patients were identified as PIK3CA mutation positive. In contrast to these two treatment responders, two additional patients identified as PIK3CA mutation positive did not respond to AZD5363 treatment. The E17 K mutation, a glutamic acid to lysine substitution, is the most common mutation in Akt1, comprising 89 % of the mutation found in this gene, and has been shown to constitutively activate Akt1, stimulating downstream signaling and transforming cells [15]. The results of the current study are consistent with findings from non-clinical studies in which AZD5363 inhibits downstream signaling of Akt1 (E17K) mutation. Moreover, the finding that Akt1-mutation-positive tumors may be sensitive to AZD5363, whereas PIK3CA-mutation-positive tumors are relatively insensitive to AZD5363, is clinically relevant [5, 15].

A phase 1 study (Study 1, D3610C00001, NCT01226316) is currently evaluating the antitumor activity of AZD5363 monotherapy in patients with tumors harboring the Akt1 mutations. Two additional studies are investigating AZD5363 in combination with endocrine therapy and chemotherapy (FAKTION study [NCT01992952] and PAKT study [NCT02423603], respectively) in patients with advanced breast cancer.

## Conclusions

In Japanese patients with advanced solid tumors, AZD5363 was generally well tolerated when given in either a continuous (bid every day) or an intermittent dosing schedule. Based on combined data from preclinical modeling, other phase 1 studies, and the current work, the 480 mg bid intermittent 4/3 dosing schedule was selected as the monotherapy dose for subsequent Phase 1b investigations. Data from these studies will provide further insight into whether AZD5363 delivers clinical benefit to patients with advanced cancer in combination with chemo- or endocrine therapy, and as monotherapy in Akt1-mutation-selected patients.
